# P-77. In vitro Activity of Phage and Vancomycin against Periprosthetic Joint Infection-Associated Staphylococcus aureus and Staphylococcus epidermidis on Orthopedic Kirschner Wires

**DOI:** 10.1093/ofid/ofaf695.306

**Published:** 2026-01-11

**Authors:** Judith Alvarez Otero, Melissa J Karau, Jayawant Mandrekar, Krupa Parmar, Kerryl Greenwood-Quaintance, Robin Patel

**Affiliations:** Mayo Clinic, Rochester, MN; Mayo Clinic, Rochester, MN; Mayo Clinic, Rochester, MN; Mayo Clinic, Rochester, MN; Mayo Clinic, Rochester, MN; Mayo Clinic, Rochester, MN

## Abstract

**Background:**

Staphylococci are the most frequent microorganisms in periprosthetic joint infection (PJI) and treatment remains challenging. Phage is being considered as a potential treatment. Here, the *in vitro* activity of phages SaMD07FSphi1or SaNSI1469phi1, alone and in combination with vancomycin against staphylococcal biofilms was evaluated.

**Figure 1:**
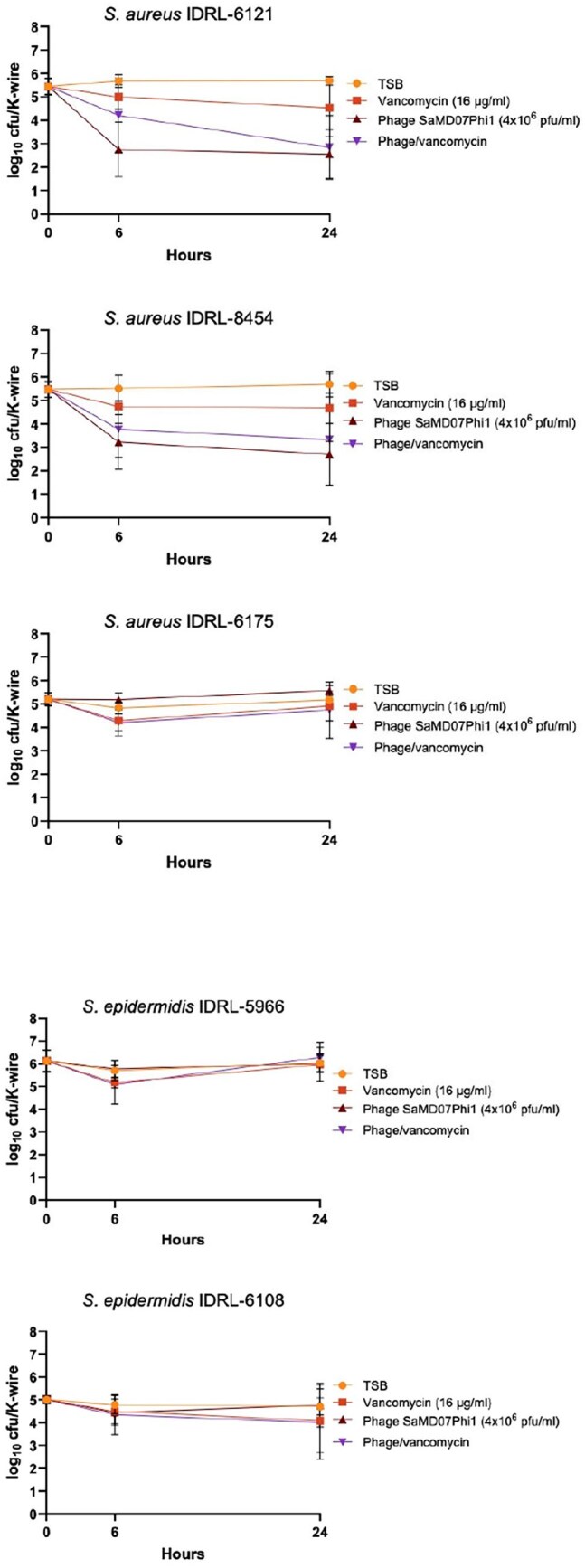
In vitro activity of phage SaMD07FSphi1 against S. aureus and S. epidermidis biofilms

**Figure 2:**
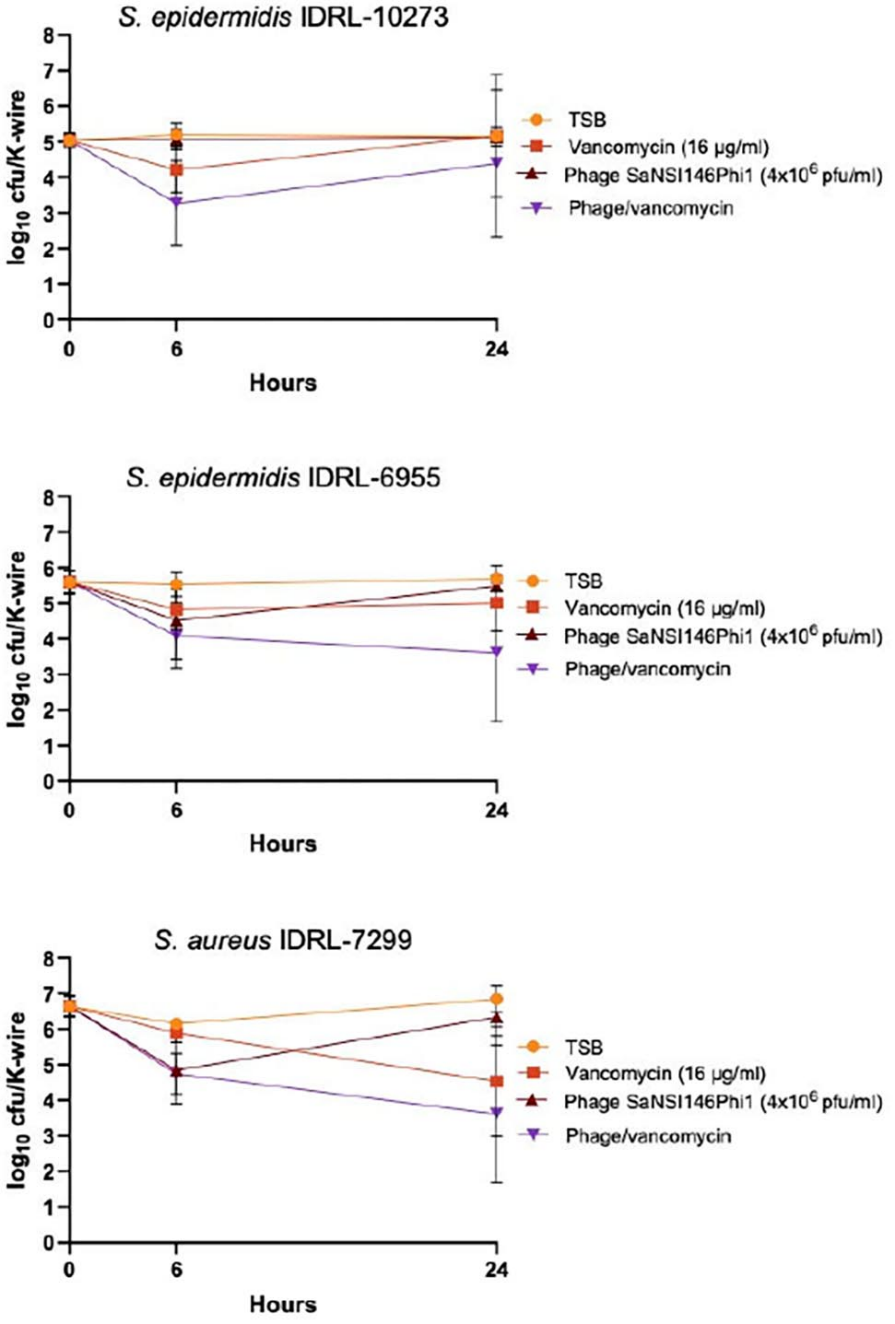
In vitro activity of phage SaNSI1469phi1 against S. aureus and S. epidermidis biofilms

**Methods:**

Biofilms were grown at 37°C on 4 cm stainless steel K-wires in tryptic soy broth (TSB) with 10^6^ cfu/ml of *S. epidermidis* or *S. aureus* (8 PJI isolates with phage activity against planktonically-grown bacteria). After 24 h, 3 K-wires were quantitatively cultured to define pre-treatment bacterial loads, with the remaining assigned to one of four treatment groups: TSB, phage (4 x 10^6^ plaque forming units/ml), vancomycin (16 µg/ml) or phage/vancomycin. K-wires were incubated for 6 or 24 h at 37°C, rinsed, sonicated, and quantitatively cultured. Testing was performed in triplicate and on 3 separate days and mean log_10_ cfu/K-wire reduction factors (LRFs) *versus* TSB calculated.

**Results:**

Phage SaMD07FSphi1 (Figure 1) plus vancomycin showed LRFs of 2.8 (p, 0.0003) and 2.3 (p, 0.0177) log_10_ cfu/K-wire at 24 h for *S. aureus* IDRL-6121 and *S. aureus* IDRL-8454, respectively, whereas phage alone showed was 3.1 (p, 0.0003) and 3.0 (p, 0.0003), respectively. All treatments were ≤1.0 log_10_ cfu/K-wire 24 h LRFs for *S. aureus* IDRL-6175, *S. epidermidis* IDRL-5966 and *S. epidermidis* IDRL-6108.This phage plus vancomycin showed LRFs of 0.6 log_10_ cfu/K-wire at 6 h for *S. epidermidis* IDRL-5966 and *S. aureus* IDRL-6175, with bacterial regrowth at 24 h. Phage SaNSI1469phi1 (Figure 2) plus vancomycin showed 24 h LRFs of 2.0 (p, 0.02) and 3.2 (p, 0.003) log_10_ cfu/K-wire for *S. epidermidis* IDRL-6955 and *S. aureus* IDRL-7299, respectively, whereas phage alone was 0.2 (p, 0.17) and 0.5 (p, 0.03) log_10_ cfu/K-wire, respectively. All treatments showed ≤1.0 log_10_ cfu/K-wire 24-hour LRFs log_10_ cfu/K-wire for *S. epidermidis* IDRL-10273. This phage alone showed 6 h LRFs of 1.0 and 1.3 for *S. epidermidis* IDRL-6955 and *S. aureus* IDRL-7299, with bacterial regrowth at 24 h.

**Conclusion:**

Phages SaMD07FSphi1 and SaNSI1469phi1 alone and with vancomycin showed activity against staphylococcal biofilms formed by 4 isolates. Increased activity was observed at 6 h in some cases, with bacterial regrowth at 24 h.

**Disclosures:**

Robin Patel, MD, a patent on Bordetella pertussis/parapertussis PCR issued, a patent on a device/method for sonication, a patent on PET imaging of bacterial infection: a patent on Bordetella pertussis/parapertussis PCR issued, a patent on a device/method for sonication, a patent on PET imaging of bacterial infection|MicuRx Pharmaceuticals and bioMérieux: Grant/Research Support|PhAST, Day Zero Diagnostics, DEEPULL DIAGNOSTICS, S.L., Nostics, HealthTrackRx, bioMérieux and CARB-X: Advisor/Consultant|Up-to-Date and the Infectious Diseases Board Review Course: Honoraria

